# C/EBPβ Regulates HIF-1α-Driven Invasion of Non-Small-Cell Lung Cancer Cells

**DOI:** 10.3390/biom15010036

**Published:** 2024-12-30

**Authors:** Seung Hee Seo, Ji Hae Lee, Eun Kyung Choi, Seung Bae Rho, Kyungsil Yoon

**Affiliations:** Cancer Metastasis Branch, Research Institute, National Cancer Center, Goyang 10408, Republic of Korea; shseo77380@ncc.re.kr (S.H.S.); jhlee5@ncc.re.kr (J.H.L.); sbrho@ncc.re.kr (S.B.R.)

**Keywords:** C/EBPβ, hypoxia, HIF-1α, metastasis, non-small-cell lung cancer

## Abstract

Metastatic cancer accounts for most cancer-related deaths, and identifying specific molecular targets that contribute to metastatic progression is crucial for the development of effective treatments. Hypoxia, a feature of solid tumors, plays a role in cancer progression by inducing resistance to therapy and accelerating metastasis. Here, we report that CCAAT/enhancer-binding protein beta (C/EBPβ) transcriptionally regulates *hypoxia-inducible factor 1 subunit alpha* (*HIF1A*) and thus promotes migration and invasion of non-small-cell lung cancer (NSCLC) cells under hypoxic conditions. Our results show that knockdown or forced expression of C/EBPβ was correlated with HIF-1α expression and that C/EBPβ directly bound to the promoter region of *HIF1A*. Silencing *HIF1A* inhibited the enhanced migration and invasion induced by C/EBPβ overexpression in NSCLC cells under hypoxia. Expression of the HIF-1α target gene, *SLC2A1*, was also altered in a C/EBPβ-dependent manner, and knockdown of *SLC2A1* reduced migration and invasion enhanced by C/EBPβ overexpression. These results indicate that C/EBPβ is a critical regulator for the invasion of NSCLC cells in the hypoxic tumor microenvironment. Collectively, the C/EBPβ-HIF-1α-GLUT1 axis represents a potential therapeutic target for preventing metastatic progression of NSCLC and improving patient outcomes.

## 1. Introduction

Lung cancer is the most frequently diagnosed cancer type and the leading cause of cancer-related deaths [1]. Non-small-cell lung cancer (NSCLC) accounts for approximately 85% of all lung cancer cases [2]. Patients with NSCLC are often diagnosed at advanced stages, typically at stage III or IV, with either locally advanced disease or metastasis to distant organs. The prognosis of metastatic NSCLC is poor, with a 5-year survival rate of approximately 7–8% [1]. Metastatic NSCLC is generally incurable, although prolonged survival can be achieved using immunotherapy, targeted therapy, and chemotherapy [3,4,5]. Understanding the metastatic process and elucidating its molecular mechanisms are crucial for the development of novel therapeutic strategies to regulate metastasis. 

Metastasis is a multi-step process including invasive migration, dissemination, and metastatic colonization [6,7], and in each step, transcriptional regulation is of importance [8]. Hypoxia is recognized as a critical driver among numerous factors that contribute to metastasis [9,10]. The rapid growth of tumors with insufficient vascular supply leads to the formation of a hypoxic tumor microenvironment (TME). This hypoxic state enhances the invasive and metastatic potential of tumor cells, promotes treatment resistance, and contributes to the development of aggressive tumors. Tumor hypoxia is associated with poor prognosis in various cancer types [11,12,13]. Consequently, targeting hypoxia-related pathways represents a promising strategy for mitigating metastasis and improving therapeutic outcomes.

The hypoxia-inducible factor 1 (HIF-1) is a key transcriptional regulator of the cellular response to low oxygen levels. HIF-1 is a heterodimer composed of an oxygen-sensitive α subunit (HIF-1α) and a constitutively expressed β subunit [14,15]. Under normoxic conditions, the α subunit is promptly degraded via the ubiquitin–proteasome pathway following hydroxylation by prolyl hydroxylases (PHDs). PHD activity is inhibited in hypoxic conditions, and HIF-1α is stabilized and dimerized with HIF-1β [14,15,16], activating a wide variety of genes involved in metabolic adaptation, drug resistance, and metastasis [11,17,18]. In addition to the post-translational regulation of HIF-1α, the transcriptional regulation of *HIF1A* is mediated by various transcription factors, coactivators, and signaling pathways, including Stat3, NF-κB, p53, Bclaf1, and p300 [10,15,19,20,21].

CCAAT/enhancer-binding protein β (C/EBPβ) is a transcription factor that has been reported to play a role in metastasis and cancer progression in various cancers. For instance, in colorectal cancer (CRC), C/EBPβ regulates the transcription of serpin family A member 1 (SERPINA1), thereby promoting migration through the activation of STAT3 signaling [22]. In addition, C/EBPβ-induced secretion of pro-inflammatory cytokines interleukin-6 (IL6) creates a positive feedback loop that activates the STAT3 signaling pathway in tumor-associated macrophages and promotes migration and invasion of lung adenocarcinoma [23]. In renal cell carcinoma, C/EBPβ also promotes migration and invasion through IL6/STAT3 signaling [24]. Elevated C/EBPβ expression has been correlated with poor prognosis and metastatic potential in triple-negative breast cancer [25]. It has been reported that the transgenic mice bearing thymidine kinase promoter containing nuclear factor IL6 (NF-IL6)/ CCAAT/enhancer binding protein β (C/EBPβ) binding site ligated to the lacZ reporter constructs displayed prominent expression of the transgene on exposure to hypoxia in lungs [26], indicating C/EBPβ responds to environmental oxygen deprivation and regulates gene expression. In human glioblastoma, C/EBPβ and Stat3 have been reported as master transcriptional regulators of mesenchymal transformation, a characteristic of tumor aggressiveness, and prognostic factors for poor clinical outcome [21]. C/EBPβ protein was elevated in hypoxic, peri-necrotic cells in GBM, as well as in hypoxia-exposed U87MG cells with concurrent induction of HIF-1α [27].

Besides C/EBPs having an important role in lung development and epithelial differentiation, C/EBPβ plays a specific role in a challenging condition such as acute phase stimuli or injury [28,29]. Therefore, we aimed to investigate the role of C/EBPβ in hypoxic responses related to lung cancer invasion, promoting metastasis. In this study, we demonstrated that C/EBPβ directly targets HIF-1α to regulate the invasiveness of NSCLC cells.

## 2. Materials and Methods

### 2.1. Cell Culture, Hypoxic Condition, and Transfection

A549 (KCLB No. 10185) and NCI-H1299 (KCLB No. 25803) cell lines were purchased from the Korean Cell Line Bank (Seoul, Republic of Korea) and cultured in RPMI 1640 medium. All cell lines were maintained in medium supplemented with 10% fetal bovine serum (FBS) and 1× penicillin-streptomycin. All cells were cultured under standard conditions at 37 °C in a humidified atmosphere with 20% O_2_ and 5% CO_2_ for normoxic conditions, and with 1% O_2_, 5% CO_2_, and 94% N_2_ for hypoxic conditions in a hypoxic chamber (Don Whitley Scientific, Bingley, UK). A549 cells were transiently transfected with either negative control siRNA (siNC) or C/EBPβ-specific siRNA (siC/EBPβ) at a final concentration of 20 nM for 24 h. Following transfection, the cells were replated according to the respective experimental conditions. The Tet-C/EBPβ plasmid was constructed in the pSBtet-GP vector (plasmid #60495, Addgene, Watertown, MA, USA), a tetracycline-inducible expression vector, using the In-Fusion^®^ HD Cloning Kit (Clontech Laboratories, Inc., Mountain View, CA, USA). Cloning primers were designed to target the SfiI enzyme site and amplify the full *CEBPB* sequence. The forward primer sequence was 5′-TTCCTACCCTCGAAAGGCCTCTGAGGCCACC ATGCAACGCCTGGTG-3′, and the reverse primer sequence was 5′-TATCGATGGAAGCTTGGCCTGACAGGCC CTAGCAGTGGCCGGA-3′. The *CEBPB* insert was amplified from pLenti-C-Myc-DDK-P2a-Puro-C/EBPbeta (RC205882L3, OriGene Technologies, Rockville, MD, USA) using KOD-Plus-Neo kit (Toyobo Co., Ltd., Osaka, Japan). The Tet-Ctrl vector was constructed by deleting the *CEBPB* insert from the Tet-C/EBPβ vector using a KOD-Plus-Mutagenesis kit (Toyobo Co., Ltd.). The constructed plasmids were transfected into NCI-H1299 cells, and the cells were sorted by flow cytometry based on the vector’s EGFP expression. NCI-H1299 cell lines containing either the Tet-Ctrl or Tet-C/EBPβ plasmids were designated as H1299-Tet-Ctrl and H1299-Tet-C/EBPβ, respectively. To induce ectopic expression of C/EBPβ in NCI-H1299 cells, 100 ng/mL doxycycline was added to the medium for 24 h. Afterward, cells were replated in doxycycline-containing medium for further experiments. Doxycycline (D9891) was purchased from Sigma-Aldrich (St. Louis, MO, USA).

### 2.2. Cell Proliferation Assay

Cells were seeded at 30% confluence in 96-well plates, and cell proliferation was assessed at 24 h intervals under normoxic or hypoxic conditions by measuring cellular ATP content using the CellTiter-Glo^®^ 2.0 Cell Viability Assay (Promega, Madison, WI, USA).

To confirm that cell migration and invasion are not related to cell proliferation, a cell viability assay was performed under the same conditions as the trans-well assay. A total of 5 × 10⁴ cells were plated in a 96-well plate with serum-free media and incubated for 24 h for A549 cells and 16 h for NCI-H1299 cells in a hypoxic chamber. Cell viability was then measured.

### 2.3. Migration and Invasion Assay

Trans-well migration and invasion assays were conducted using trans-well chambers (Corning Incorporated, Corning, NY, USA). For the invasion assay, the chambers were pre-coated with 0.5 µg/mL Matrigel in 24-well plates (Corning Incorporated). A cell suspension (5 × 10⁴ cells/mL) in serum-free medium was added to the upper chamber, while 700 μL of medium containing 10% fetal bovine serum was added to the lower chamber. The chambers were incubated under hypoxic conditions at 37 °C for 24 h (A549) or 16 h (NCI-H1299). As a normoxic control, cells were incubated under identical conditions in a normoxic environment. After incubation, cells remaining in the upper chamber were gently removed using a cotton swab. Invaded cells were fixed with 4% paraformaldehyde for 10 min and stained with 0.1% crystal violet for 20 min at room temperature. Cells beneath the membrane were scanned using a Vectra^®^ polarisTM (AKOYA bioscience, Marlborough, MA, USA), and the number of migrated or invaded cells was quantified by calculating the average from three random fields of view per well at 10x magnification using ImageJ software (version 1.54a).

### 2.4. Tissue Microarray (TMA) Analysis

Lung cancer tissue array (Cat. No. CCA4) was obtained from Superbiochips Laboratories (Seoul, Republic of Korea), and tissue microarray analysis was commercially commissioned from Superbiochips Laboratories. The CCA4 slides consist of 10 adjacent normal lung tissues, 49 primary, 10 metastatic lung cancers, and 1 carbon control. Metastatic tumor samples and adjacent normal matching with primary tumor were 10 and 2, respectively. One primary tumor sample was missing during the tissue processing, and therefore, nine sets of primary and matching metastatic tumors were analyzed to compare C/EBPβ protein expression. The section was stained with C/EBPβ antibody (1:30, sc-150, Santacruz Biotechnology, Inc., Dallas, TX, USA) with the Ventana BenchMark XT Staining systems (Ventana Medical Systems, Inc., Tucson, AZ, USA). The C/EBPβ expression was scored from 0 to 4 based on the intensity and percentage of positive staining of cancer cells in the tissue field by two medical pathologists independently.

### 2.5. Microarray and Bioinformatics Analysis

A549 cells were transiently transfected with either negative control siRNA (siNC) or C/EBPβ-specific siRNA (siC/EBPβ) at a final concentration of 20 nM for 24 h. Then, cells were cultured under both normoxic and hypoxic conditions for 4 h. Total RNA was extracted using TRIzol reagent (Invitrogen, Carlsbad, CA, USA) following the manufacturer’s protocol. Microarray using Agilent’s Gene Expression Hybridization Kit was commercially commissioned by Genomic tree, Inc. (Daejeon, Republic of Korea). Differentially expressed genes (DEGs) were extracted from microarray data through a two-step filtering process: the first filter based on normoxia versus hypoxia, and the second filter based on control versus C/EBPβ knockdown. Gene with more than |log_2_FC| > 0.5 were selected. A total of 2115 DEGs were subjected to Gene ontology (GO) and Kyoto Encyclopedia of Gene and Genome (KEGG) analyses.

### 2.6. Reverse Transcription–Quantitative Polymerase Chain Reaction (RT-qPCR)

Total RNA was extracted using the AccuPrep^®^ Universal RNA Extraction Kit (Bioneer Inc, Daejeon, Republic of Korea), and cDNA was synthesized with the Dyne RT Dry MIX Kit (DYNE Bio, Gyeonggi, Republic of Korea). Real-time polymerase chain reaction (PCR) was conducted on the LightCycler^®^ 96 Real-Time PCR System (Roche, Basel, Switzerland). Each reaction utilized 20 ng of cDNA in conjunction with SYBR Green. Data are presented as fold change in each target gene expression, normalized to *HPRT* expression. The primer sequences for PCR were as follows: *CEBPB* (F: 5′-GGAGACGCAGCACAAGGT-3′ and R: 5′-AGCTGCTTGAACAAGTTCCG-3′), *HIF1A* (F: 5′-TCATCCAAGAAGCCCTAACGTG-3′ and R: 5′-TTTCGCTTTCTCTGAGCATTCTG-3′), *SLC2A1* (F: 5′-GCCTGAGACCAGTTGAAAGCAC-3′ and R: 5′-CTGCTTAGGTAAAGTTACAGGAG-3′) and *HPRT* (F: 5′-GCTATAAATTCTTTGCTGACCTGCTG-3′ and R: 5′-AATTACTTTTATGTCCCCTGTTGACTGG-3′). The PCR protocol included: an initial denaturation at 95 °C for 5 min, followed by 40 cycles of 94 °C for 10 s, 60 °C for 10 s, and 72 °C for 10 s, with a final extension step at 72 °C for 9 min.

### 2.7. Western Blot Analysis

Whole-cell lysates were prepared using radioimmunoprecipitation assay (RIPA) buffer containing a protease inhibitor cocktail, phosphatase inhibitors (Calbiochem, San Diego, CA, USA), phenylmethylsulfonyl fluoride (PMSF), and dithiothreitol (DTT). Protein concentrations were quantified using the Micro Bicinchoninic Acid (BCA) Protein Assay Kit (Thermo Scientific, Rockford, IL, USA). Equal amounts of protein were separated by 10–15% sodium dodecyl sulfate–polyacrylamide gel electrophoresis (SDS-PAGE) and transferred onto polyvinylidene difluoride (PVDF) membranes (BioRad, Hercules, CA, USA) using a wet transfer system (BioRad, Hercules, CA, USA). The following antibodies were utilized: C/EBPβ (Santa Cruz, sc-7962), β-actin (Santa Cruz, sc-477778), PCNA (Santa Cruz, sc-56) (all from Santacruz Biotechnology, Dallas, TX, USA); and HIF1α (CST, #3716), and GAPDH (CST, #2118), phospho-AKT (CST, #4060), AKT (CST, #9272), phospho-mTOR (CST, #5536), mTOR (CST, #2983), Raptor (CST, #2280), phospho-p70S6K(CST, #9208), p-S6 (CST, #4858), phospho-4EBP1 (CST, #2855) (all obtained from Cell Signaling Technology, Inc., Danvers, MA, USA).

### 2.8. Chromatin Immunoprecipitation Analysis

The ChIP-on-chip assay data from our previous study [30] was reanalyzed to identify C/EBPβ binding region on the *HIF1A* promoter. Public ChIP-seq data against C/EBPβ (ID: SRX190327) and H3K27ac (ID: SRX10976170) were downloaded from ChIP Atlas. Using the JASPAR database, potential C/EBPβ binding sites were predicted. To validate these predictions, chromatin immunoprecipitation (ChIP) assay was performed using the EZ-ChIP assay (Upstate Biotechnology, Lake Placid, NY, USA) following the manufacturer’s protocol. Briefly, A549 cells were cultured under both normoxic and hypoxic conditions for 4 h, and 10 ug sheared chromatin was used for immunoprecipitation with C/EBPβ antibody (2 ug, sc-150X, Santa Cruz) or normal rabbit IgG (2 ug, #2729, CST). C/EBPβ binding regions on the *HIF1A* promoter were analyzed by quantitative real-time PCR (qRT-PCR). Primers for PCR analysis were as follows: S1 (F, 5′-TTTTGAACAGAGAGCCCAGCA-3′ and R, 5′-GAGAAGGGATTTCGGTTGCC-3′); S2 (F, 5′-AGAGTGCGGTGGGTGACATT-3′ and R, 5′-AAAAAGCAGACTTCGCCTCG-3′).

### 2.9. Statistical Analysis

All data points are presented as the means ± standard deviations (SD) from three independent experiments conducted in triplicate. Comparisons between two groups were analyzed using Student’s *t*-test, while comparisons involving more than two groups were performed using two-way ANOVA. Statistical significance was set at *p* < 0.05.

To determine the statistical power of the TMA study, we performed a power analysis using the G*Power software (version 3.1.9.7). The analysis was based on the following parameters: an effect size (Cohen’s *d*) of −1.42, a significance level (*α*) of 0.05, and a power (1 − *β*) of 0.8. The calculated power for our sample size confirmed that the study design was adequate to detect the observed effect sizes between the primary tumor and metastatic tumor groups.

Disease-free survival and correlation analyses were performed using gene expression profiling interactive analysis (GEPIA). This platform was used to analyze disease-free survival and investigate the correlation between *HIF1A*, *CEBPB*, or *SLC2A1* expression in patients with lung adenocarcinoma.

## 3. Results

### 3.1. C/EBPβ Increases Proliferation, Migration, and Invasion in NSCLC Cells Under Hypoxic Conditions

**Figure 1:**
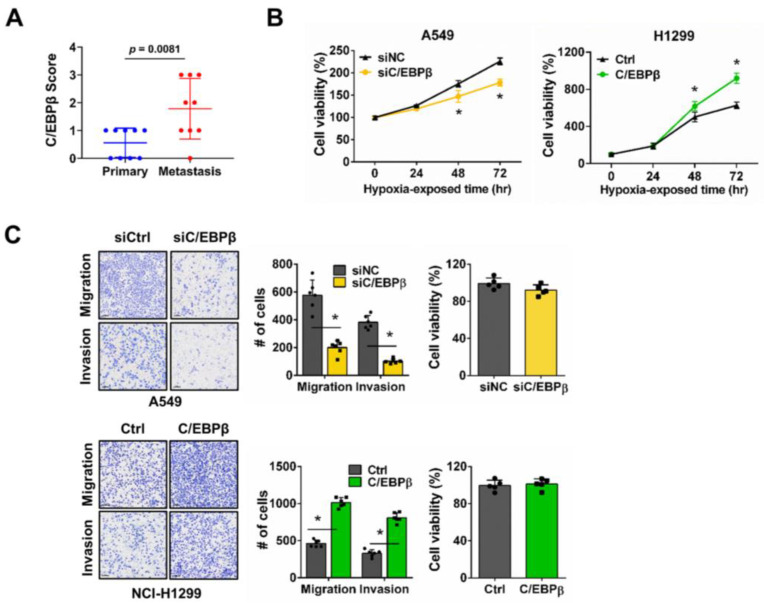
CCAAT/enhancer-binding protein β (C/EBPβ) regulates non-small-cell lung cancer (NSCLC) cell proliferation, migration, and invasion under hypoxic conditions: (**A**) Immunohistochemistry (IHC) score for C/EBPβ between the primary and matched metastatic tumors. Data are presented as the mean ± SD. Statistical significance was determined using the *t*-test (*p* = 0.0081). Power analysis was performed using G*Power software. The analysis confirmed that the sample size was sufficient to detect the observed effect size (Cohen’s *d* = −1.42) with a significance level (*α*) of 0.05 and a power (1 − *β*) of 0.8096. Power (1 − *β*) represents the probability of rejecting a false null hypothesis. (**B**) Cell proliferation was evaluated at 24 h intervals under hypoxic conditions. Data are presented as the mean ± SD from independent experiments performed in triplicate. * *p* < 0.05 vs. each control. (**C**) Trans-well migration and invasion assays were performed under hypoxic conditions using the A549 (top) and NCI-H1299 (bottom) cell lines. Scale bar, 100 μm. Cell proliferation assays were conducted under the same conditions as those used for the trans-well assay. Data are presented as the mean ± SD from three independent experiments. * *p* < 0.05 vs. each control.

To investigate the clinical association of C/EBPβ with metastasis, C/EBPβ expression was compared between primary and matched metastatic lung cancer tissues. The pathological classification, staging, and metastatic sites of the nine patients from the tissue microarray are shown in Table 1. The staining images of nine sets of primary and metastatic lung cancer tissues, along with their respective staining scores, are provided in Figure S1. C/EBPβ protein levels were significantly higher in metastatic tumors, suggesting that C/EBPβ may play a role in the metastatic process (Figure 1A). To evaluate the potential role of C/EBPβ in metastasis, we conducted an experimental metastasis analysis via mouse tail vein injection [31,32]. C/EBPβ knockdown resulted in a significant reduction in tumor foci formation in the lungs, presented by a decrease in lung weight compared with the control groups (Figure S2). Hypoxia in the tumor microenvironment is a key factor that facilitates cancer metastasis by triggering a cascade of molecular and cellular changes [9,11]. In our study, we investigated the role of C/EBPβ in regulating proliferation in NSCLC cell lines with different C/EBPβ levels, A549 and NCI-H1299, under hypoxic conditions (Figure S3). Previously, we reported that the proliferation of NSCLC cells, including A549 and NCI-H1299, was inhibited by C/EBPβ knockdown in normoxia [30]. Under hypoxic conditions, C/EBPβ knockdown significantly suppressed A549 cell proliferation (Figure 1B and Figure S4A). Caspase-7 cleavage was not observed in C/EBPβ knockdown cells under hypoxia, confirming that decreased cell viability was due to reduced proliferation (Figure S4C). Conversely, C/EBPβ overexpressing NCI-H1299 cells exhibited increased proliferation (Figure 1B and Figure S4B). When A549 cells were exposed to hypoxia, elongation of the cells was observed, which was quantitatively reflected by a decrease in circularity and an increase in the aspect ratio (AR). In contrast, when C/EBPβ was knocked down under hypoxic conditions, stronger cell–cell interactions were observed, leading to an epithelial-like cell shape. This was supported by an increase in circularity and a decrease in AR (Figure S5). These results suggest that C/EBPβ plays a role in regulating the proliferation and motility of NSCLC cells under hypoxic conditions. Furthermore, migration and invasion assays were performed to examine the dependency of the motile phenotype on C/EBPβ expression. In A549 cells, C/EBPβ silencing significantly decreased migration and invasion under hypoxic conditions. Conversely, migration and invasion were significantly enhanced in C/EBPβ overexpressing NCI-H1299 cells (Figure 1C). Cell proliferation and PCNA levels remained unchanged under the confluent conditions without serum used for the migration and invasion assays (Figure S6), confirming that changes in cell migration and invasion between groups are not a result of the changes in cell viability.

In summary, C/EBPβ expression is significantly associated with metastasis and serves a pivotal role in modulating proliferation and invasiveness in NSCLC cells under hypoxic conditions.

### 3.2. C/EBPβ Regulates HIF1A Expression by Directly Binding to Its Promoter

**Figure 2:**
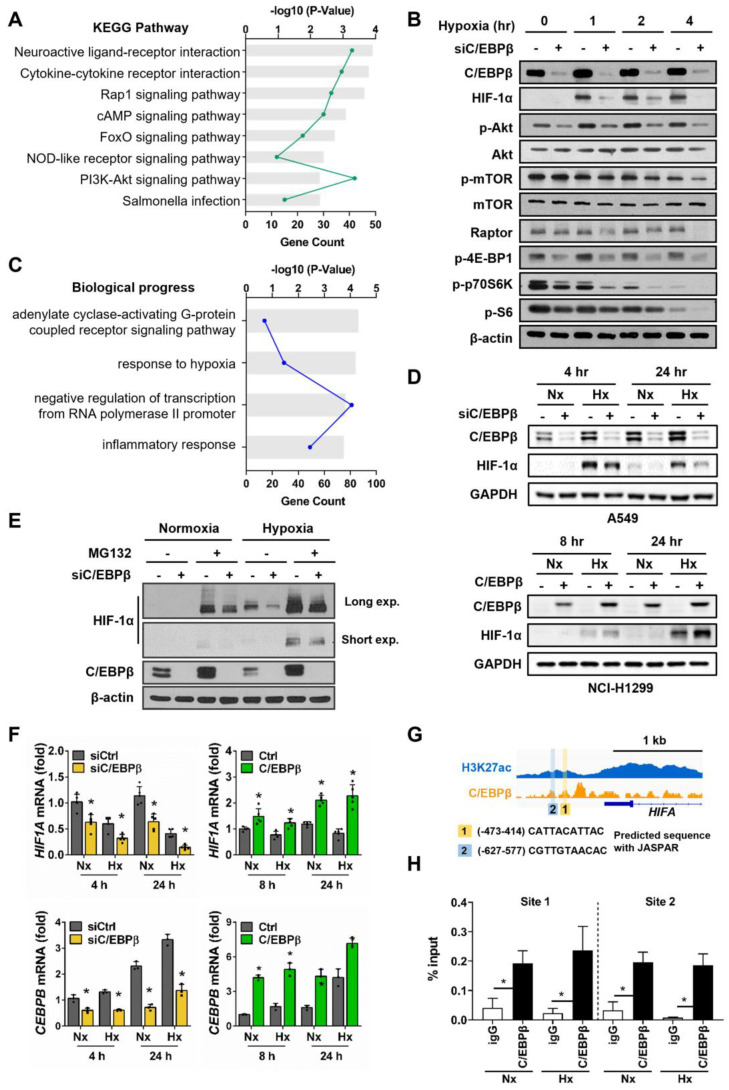
C/EBPβ transcriptionally regulates *hypoxia-inducible factor 1 subunit alpha* (*HIF1A*) under hypoxic conditions: (**A**) A total of 2115 DEGs identified under both hypoxic and *CEBPB* knockdown conditions were used for the KEGG pathway analysis. Pathways with *p* < 0.01 are represented. (**B**) Protein levels related to AKT/mTOR signaling were examined by western blot analysis. A549 cells were transfected with negative control siRNA (siNC) or siC/EBPβ and then incubated under hypoxic conditions for the indicated times. β-actin was used as the loading control. (**C**) A total of 2115 DEGs identified under both hypoxic conditions with *CEBPB* knockdown were used for GO analysis. Pathways with *p* < 0.001 are presented. (**D**) HIF-1α protein levels based on C/EBPβ expression were analyzed using western blotting. A549 and NCI-H1299 cells were maintained under hypoxic conditions for specific time periods. GAPDH was used as a loading control. (**E**) HIF-1α protein levels were analyzed using western blotting. A549 cells were pretreated with 10 μM MG-132 and incubated under hypoxic conditions for 4 h. (**F**) *CEBPB* and *HIF1A* mRNA levels were measured using RT-qPCR. C/EBPβ-silenced A549 cells and H1299-Tet-C/EBPβ cells were subjected to hypoxic conditions for specific time periods. Data are presented as the mean ± SD from three independent experiments. * *p* < 0.05 vs. each control (**G**) Chromatin immunoprecipitation sequencing (ChIP-seq) data for the *HIF1A* gene were collected and presented through a graph. H3K27ac served as positive controls for the active promoters. A schematic diagram was constructed based on the JASPAR results, highlighting the C/EBPβ binding sites (labeled 1 and 2). C/EBPβ binding sequence predicted by JASPAR is represented. (**H**) The extent of C/EBPβ binding was quantified using qPCR with primers specific to each of these binding sites. Original images of (**B**,**D**,**E**) can be found in Supplementary Materials.

We conducted Kyoto Encyclopedia of Genes and Genomes (KEGG) pathway and Gene Ontology (GO) analyses of differentially expressed genes (DEGs) extracted from microarray data and identified through a two-step filtering process to select a hypoxia-responsive C/EBPβ-dependent gene set: the first filter based on normoxia versus hypoxia, and the second filter based on control versus C/EBPβ knockdown. KEGG pathway analysis identified the PI3K-Akt signaling pathway (Figure 2A). Phosphorylation of Akt and mTOR was reduced upon C/EBPβ knockdown in hypoxia, and notably, the levels of Raptor, a key component of mTORC1, were also decreased (Figure 2B). Raptor recruits substrate proteins, such as 4E-BP1 and p70 S6K, to mTORC1, facilitates their phosphorylation, and regulates protein translation [33]. C/EBPβ inhibition resulted in a decrease in Raptor, which subsequently led to reduced phosphorylation of mTORC1 substrate proteins, p70S6K and 4E-BP1. Moreover, the phosphorylation of the ribosomal protein S6 (S6), a well-known substrate of p70S6K, also decreased. Unexpectedly, phospho-AKT, Raptor, phospho-p70 S6K, and phospho-4E-BP1 levels were also decreased with C/EBPβ knockdown in normoxic conditions. These findings suggest that C/EBPβ may play a role in regulating protein translation via the Akt/mTOR signaling pathway.

GO terms related to biological processes with *p*-values < 0.001 are shown in Figure 2C. The GO term response to hypoxia ranked second, and hypoxia-inducible factor 1 subunit alpha (*HIF1A*) was included. HIF-1α is a key transcription factor that regulates over 100 genes involved in various biological processes, including invasion and metastasis [34]. Therefore, we explored the relationship between C/EBPβ and *HIF1A* under different oxygen concentrations. In A549 cells, hypoxia induced the expression of HIF-1α, which was reduced by C/EBPβ knockdown, and in NCI-H1299 cells, hypoxia-induced expression of HIF-1α was enhanced by C/EBPβ overexpression, suggesting that C/EBPβ is involved in the regulation of HIF-1α expression (Figure 2B,D). Next, we investigated the mechanism through which C/EBPβ regulates HIF-1α under hypoxic conditions. Under hypoxia, HIF-1α protein levels increased in control cells compared with C/EBPβ knockdown cells instantly, an hour after exposure to hypoxia cells (Figure 2B). The half-life of HIF-1α is very short, and it is primarily degraded via the ubiquitin–proteasome pathway mediated by the von Hippel–Lindau (VHL) under normoxic conditions [14]. Thus, we first examined whether C/EBPβ regulates the protein stability of HIF-1α. To determine whether C/EBPβ regulates HIF-1α at the post-translational level, we utilized the proteasome inhibitor MG132 and found that HIF-1α expression increased, reaffirming the well-established fact that HIF-1α undergoes proteasome-dependent degradation (Figure 2E). Under hypoxia, the dramatic difference in HIF-1α protein levels between control and C/EBPβ- silenced A549 cells was observed. In the presence of MG132, C/EBPβ knockdown cells kept displaying lower levels of HIF-1α compared with control cells under hypoxic conditions, suggesting that C/EBPβ is unlikely to regulate the proteasomal degradation pathway of HIF1α. Under normoxic conditions, the reduction in HIF-1α levels in C/EBPβ knockdown cells was observed in the presence of MG132, reinforcing that C/EBPβ regulates *HIF1A* expression rather than the protein stability of HIF-1α.

To determine whether the altered HIF-1α protein expression dependent on C/EBPβ is regulated at the transcriptional level, we examined *HIF1A* mRNA levels. Microarray analysis confirmed alterations in *HIF1A* mRNA levels upon *CEBPB* inhibition (Figure S7). Consistent with protein expression, *HIF1A* mRNA levels were either downregulated or upregulated in response to changes in C/EBPβ expression in normoxia and hypoxia (Figure 2F), indicating that C/EBPβ regulates *HIF1A* transcript levels, irrespective of oxygen concentration.

Based on ChIP-on-chip assay data, we identified C/EBPβ binds to the *HIF1A* promoter regions. Using the JASPAR database, two potential C/EBPβ binding sites were predicted with 627 and 414 bp upstream of the transcription start site (Figure 2G). Additionally, these binding regions were predicted to be active promoter regions through H3K27ac ChIP-seq (Figure 2G). To confirm C/EBPβ binding at sites 1 and 2 on the *HIF1A* promoter, we performed a ChIP assay and found that significant binding of C/EBPβ was observed at all sites on the *HIF1A* promoter under both normoxic and hypoxic conditions (Figure 2H). This sustained binding indicates the potential for continuous regulation of *HIF1A* by C/EBPβ, irrespective of oxygen levels.

In summary, under normoxic and hypoxic conditions, *HIF1A* mRNA levels remain consistently maintained, suggesting that C/EBPβ continuously regulates *HIF1A* transcription. Although direct observation of protein changes was challenging under normoxic conditions due to rapid degradation, under hypoxic conditions, C/EBPβ knockdown led to a decrease in *HIF1A* mRNA levels, which was accompanied by a corresponding reduction in protein levels. Since C/EBPβ does not significantly influence HIF-1α degradation, changes in mRNA levels seem to reflect changes in protein levels. These results indicate that C/EBPβ plays a crucial role in regulating HIF-1α expression at the transcriptional level by directly binding to its promoter, thereby influencing the invasiveness of NSCLC cells. 

### 3.3. SLC2A1 Regulates Migration and Invasion of NSCLC Cells Under Hypoxic Conditions, Downstream of C/EBPβ-HIF-1α Axis

**Figure 3:**
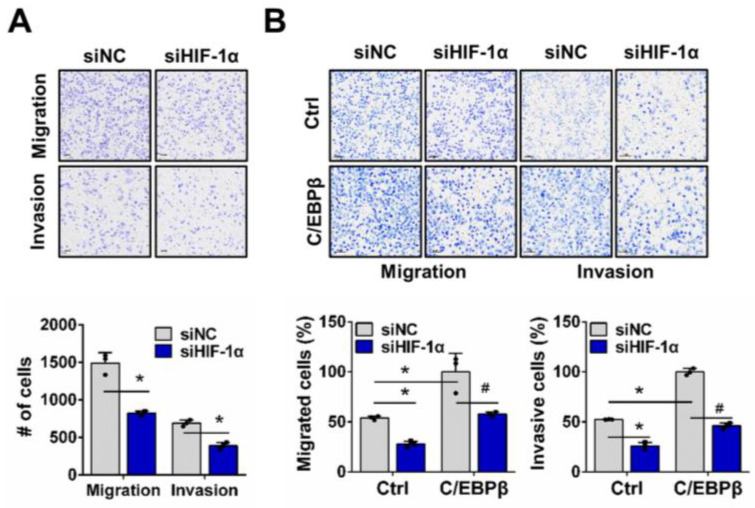
HIF-1α regulates C/EBPβ-induced migration and invasion of NSCLC cells in hypoxia. (**A**,**B**) Effects of HIF-1α downregulation on migration and invasion were evaluated in A549 and H1299-Tet-C/EBPβ cells using a tans-well assay: (**A**) A549 cells were exposed to hypoxic conditions for 24 h. (**B**) NCI-H1299 cells were maintained under hypoxic conditions for 16 h. Data are presented as the mean ± SD from three independent experiments. Scale bar, 100 μm. * *p* < 0.05 vs. each control, # *p* < 0.05 vs. C/EBPβ with siNC in NCI-H1299.

We examined whether C/EBPβ-driven migration and invasion under hypoxic conditions occur through a HIF-1α-dependent mechanism, utilizing a trans-well assay. When we evaluated the control of migration and invasion in response to HIF-1α depletion in A549 cells with high C/EBPβ expression, we confirmed that HIF-1α depletion reduced migration and invasion of A549 cells, consistent with previous reports [35,36,37] (Figure 3A and Figure S8A). However, silencing HIF-1α did not affect the migration and invasion of A549 cells under normoxia (Figure S9A). Then, we investigated whether HIF-1α is involved in the increased migration and invasion driven by C/EBPβ and found that deficiency of HIF-1α in C/EBPβ overexpressing NCI-H1299 cells decreased migration and invasion to similar levels shown in the NCI-H1299 control group (Figure 3B and Figure S8B). Overexpression of C/EBPβ under normoxic conditions increased migration and invasion, whereas silencing HIF-1α did not reduce migration and invasion, as observed under hypoxic conditions (Figure S9B). In addition, proliferation, which was increased by C/EBPβ, significantly decreased to a level similar to the control under hypoxia when HIF-1α was knocked down. However, this effect was not observed under normoxic conditions (Figure S9C). Thus, these results indicate that HIF-1α mediates C/EBPβ-induced migration and invasion of NSCLC cells under hypoxic conditions.

**Figure 4:**
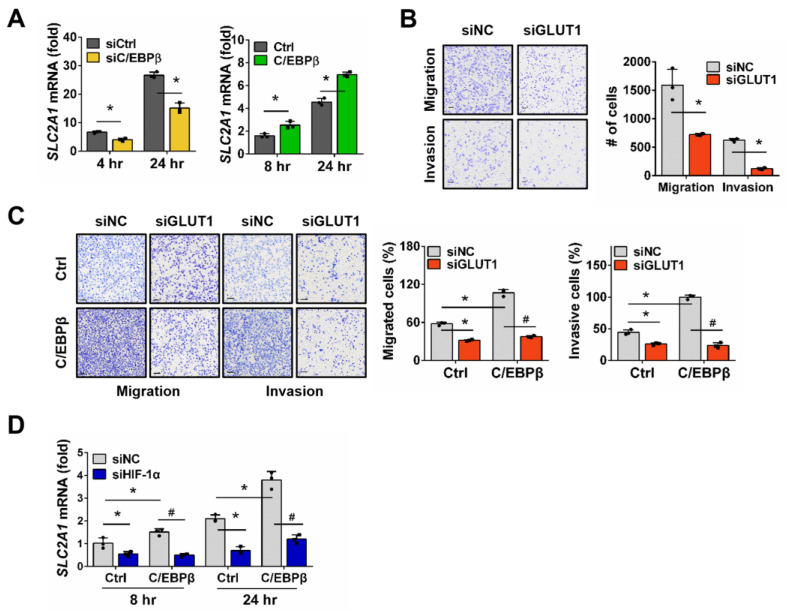
Migration and invasion of NSCLC cells are increased through the C/EBPβ-HIF-1α- Glucose transporter 1 (GLUT1) axis under hypoxic conditions: (**A**) *Solute carrier family 2, facilitated glucose transporter member* (*SLC2A1*) mRNA levels were measured using RT-qPCR in C/EBPβ-silenced A549 and H1299-Tet-C/EBPβ cells after exposure to hypoxic conditions for the specific periods. Data are presented as the mean ± SD from three independent experiments. * *p* < 0.05 vs. each control (**B**,**C**) The impact of *SLC2A1* downregulation on migration and invasion was assessed in A549 and H1299-Tet-C/EBPβ cells using the trans-well assay, following the same experimental methodology described in Figure 3A,B. Data are presented as the mean ± SD from three independent experiments. Scale bar, 100 μm. * *p* < 0.05 vs. each control, # *p* < 0.05 vs. C/EBPβ with siNC in NCI-H1299. (**D**) mRNA levels of *SLC2A1* were quantified using RT-qPCR. H1299-Tet-C/EBPβ cells were transfected with siNC or siHIF-1α and exposed to hypoxia for the specified durations. Data are presented as the mean ± SD from three independent experiments. * *p* < 0.05 vs. control at each group; # *p* < 0.05 vs. C/EBPβ with siNC at each time.

Among the HIF-1α target genes associated with migration and invasion of cancer cells, our microarray data showed that *solute carrier family 2, facilitated glucose transporter member* (*SLC2A1*) mRNA exhibited a significant increase in response to hypoxia (Figure 4A and Figure S7). Glucose transporter 1 (GLUT1), a single transporter protein encoded by the *SLC2A1* gene, promotes glucose uptake and enhances glycolysis in cancer cells [38]. We then evaluated the changes in *SLC2A1* mRNA levels in response to C/EBPβ expression under hypoxic conditions. Knockdown of C/EBPβ in A549 cells significantly reduced *SLC2A1* expression, while forced expression of C/EBPβ in NCI-H1299 cells significantly increased *SLC2A1* expression. Trans-well assays were performed to examine whether *SLC2A1* regulates cell migration and invasion. Silencing *SLC2A1* in A549 cells led to a dramatic reduction in both migration and invasion. It has been previously reported that *SLC2A1* is involved in migration and invasion in various NSCLC cell lines [39,40]. Consistent with these findings, we experimentally confirmed that the reduction in *SLC2A1* expression in A549 cells led to a decrease in migration and invasion (Figure 4B and Figure S8C). Additionally, C/EBPβ-induced migration and invasion were completely abolished by *SLC2A1* knockdown (Figure 4C and Figure S8D). To determine whether these results were due to a HIF-1α-mediated response, we silenced *HIF1A* in C/EBPβ overexpressing NCI-H1299 cells and examined the expression of *SLC2A1*. We observed that the expression of *SLC2A1*, which was upregulated by C/EBPβ, was reduced when HIF-1α expression was inhibited under hypoxia (Figure 4D and Figure S8E). However, under normoxia, *SLC2A1* levels did not change following C/EBPβ overexpression or HIF-1α silencing (Figure S8F). These results suggest that *SLC2A1* is regulated by HIF-1α in a C/EBPβ-dependent manner under hypoxia.

### 3.4. CEBPB, HIF1A, and SLC2A1 Are Associated with Prognosis of Lung Adenocarcinoma Patients

**Figure 5:**
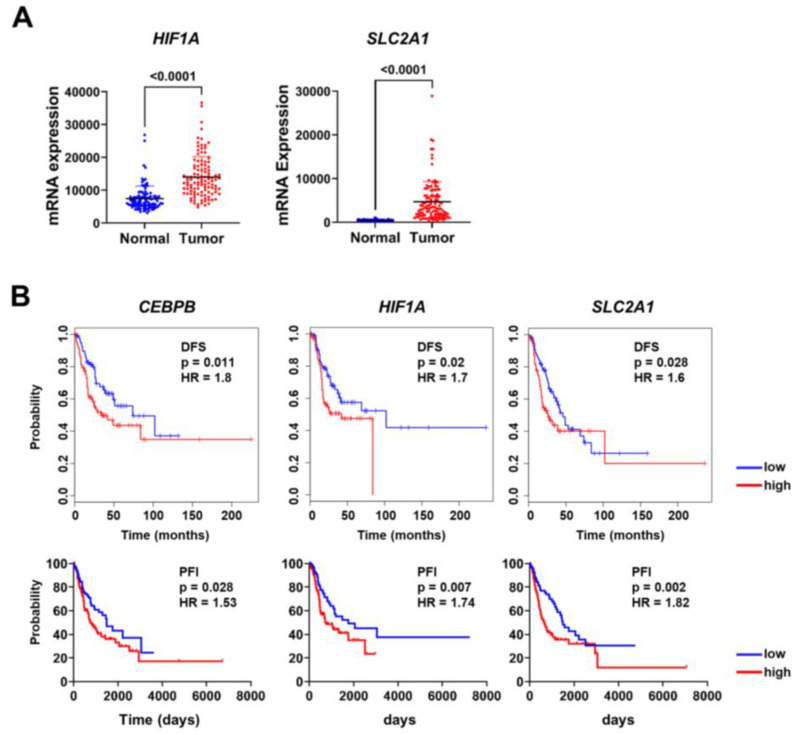
*HIF1A* and *SLC2A1* expression is associated with prognosis in patients with lung adenocarcinoma: (**A**) The expression levels of *HIF1A* and *SLC2A1* were compared between lung adenocarcinoma and normal lung tissues using data from the CPTAC database. (**B**) *CEBPB*, *HIF1A*, and *SLC2A1* were analyzed for their association with disease-free survival (DFS) and progression-free interval (PFI) in patients with lung adenocarcinoma. DFS was analyzed using GEIPA, and PFI was analyzed using UCSC Xena. HR and *p*-values are displayed on each plot.

We performed an expression analysis of *HIF1A*, and *SLC2A1* in lung adenocarcinoma using the CPTAC dataset. *HIF1A* and *SLC2A1* expression was significantly higher in lung adenocarcinoma tissues than in adjacent normal tissues (Figure 5A). *HIF1A* expression was correlated with *CEBPB* and *SLC2A1* in lung adenocarcinoma (Figure S10).

Additionally, survival analysis was performed to confirm the relationship between the expression levels of *CEBPB*, *HIF1A*, and *SLC2A1* and clinical outcomes. Using the TCGA dataset, we analyzed both disease-free survival (DFS) and progression-free interval (PFI). Patients with higher *CEBPB*, *HIF1A*, and *SLC2A1* showed shorter DFS and PFI, suggesting a potential association between elevated *CEBPB*, *HIF1A*, and *SLC2A1* expression and less favorable outcomes (Figure 5B). Previous studies have reported *SLC2A1* as a prognostic marker in various cancers, including NSCLC [39,41,42]. Building on this knowledge, our study investigates the mechanistic regulation of *SLC2A1* via the C/EBPβ-HIF-1α axis under hypoxic conditions. These findings provide additional context for the relevance of C/EBPβ in NSCLC.

In conclusion, our results show that HIF-1α mediates C/EBPβ-induced migration and invasion of NSCLC cells in hypoxia. As a downstream target of HIF-1α, *SLC2A1* plays a crucial role in this process.

## 4. Discussion

As tumors grow, their microenvironment becomes hypoxic, triggering cancer cells to activate adaptive responses that regulate genes involved in survival, angiogenesis, metabolic reprogramming, invasion, and metastasis [9]. Numerous studies have demonstrated that HIF-1α regulates these processes as a central player in tumor progression [43,44,45,46,47]. Notably, a meta-analysis conducted on 17 NSCLC histological studies found that HIF-1α expression was significantly higher in lung cancer tissues compared with normal tissues and was more elevated in NSCLC patients than in small-cell lung cancer patients [48]. Furthermore, tissues with lymph node metastasis showed higher HIF-1α expression than tissues without lymph node metastasis [49]. Notably, NSCLC patients with positive HIF-1α expression in their tumor tissues had lower overall survival rates compared with those with negative HIF-1α expression [50]. In the present study, we demonstrated that C/EBPβ transcriptionally regulates *HIF1A* in the hypoxic microenvironment of NSCLC (Figure 2G,H), emphasizing C/EBPβ as a key regulator of migration and invasion.

The PI3K/AKT/mTOR pathway promotes HIF-1α translation under normoxia by phosphorylating 4E-BP1 and S6 kinase, promoting cap-dependent translation. This mechanism ensures robust HIF-1α protein production in response to growth factors, cytokines, or oncogenic signals [14,51]. On the other hand, under hypoxic conditions, mTOR activity is inhibited to conserve cellular energy, leading to a global reduction in protein translation. However, HIF-1α translation is selectively maintained through cap-independent mechanisms, such as internal ribosome entry sites (IRES) or hypoxia-induced RNA-binding proteins [51,52,53,54]. This phenomenon supports our result that HIF-1α expression remained stable despite the gradual decrease in the expression of p-4E-BP1 and p-S6, which are involved in translation, following hypoxia exposure (Figure 2B). Additionally, although the translation mechanism of HIF-1α changes during hypoxia, the decrease in HIF-1α expression in C/EBPβ knockdown cells suggests that C/EBPβ regulates HIF-1α through a different mechanism, independent of translational regulation. Furthermore, when MG132 was administered, we observed a reduction in HIF-1α accumulation due to C/EBPβ knockdown, not only under hypoxic but also under normoxic conditions. This suggests that C/EBPβ is involved in the transcriptional regulation of HIF-1α in both normoxia and hypoxia (Figure 2E). We demonstrated this through changes in mRNA levels by regulating C/EBPβ expression and ChIP analysis in normoxia and hypoxia (Figure 2F,G).

Metabolic reprogramming enables cancer cells to adapt to various stages of the metastatic process, allowing them to survive in challenging microenvironments that meet their increased demand for nutrients and energy [55]. Unlike normal cells, cancer cells primarily metabolize glucose via aerobic glycolysis, a phenomenon known as the Warburg effect [55]. Under prolonged hypoxic conditions, HIF-1α upregulates the expression of glucose transporters such as GLUT1. Increased *SLC2A1* expression has been associated with poor survival outcomes in various cancers [56,57,58,59]. Specifically, elevated *SLC2A1* expression is associated with poor overall survival in pancreatic adenocarcinoma, where it promotes cell proliferation, invasion, and metastasis [56,57]. High *SLC2A1* levels in patients with CRC have been correlated with an increased incidence of lymph node metastasis and mortality [58,59]. Additionally, from an analysis of a public database, we showed that *SLC2A1* expression was significantly higher in lung adenocarcinoma compared with adjacent normal tissues (Figure 5A). Furthermore, patients with high *SLC2A1* expression had shorter disease-free survival rates and progression-free intervals than those with low expression (Figure 5B). This provides additional insight into the role of *SLC2A1* in NSCLC progression, particularly in the context of the hypoxic tumor microenvironment.

## 5. Conclusions

Our study demonstrated that C/EBPβ plays a pivotal role in regulating cell morphology, migration, and invasion under hypoxic conditions. Mechanistically, C/EBPβ transcriptionally controls HIF-1α expression and thus modulates HIF-1α-driven *SLC2A1* expression in hypoxia. Given the critical role of GLUT1 in cancer cell metabolism, essential for metastasis, our findings suggest that the C/EBPβ-HIF-1α-GLUT1 axis serves as a potential target for lung cancer treatment.

## Figures and Tables

**Table 1:** Clinicopathological features of patients with lung cancer.

No.	Diagnosis	Stage	T	N	Metastatic Site
1	squamous cell carcinoma	IIIB	4	2	Lymph node
2	squamous cell carcinoma	IIIA	2	2	Lymph node
3	squamous cell carcinoma	IIA	2	1	Lymph node
4	adenocarcinoma	IB	2	0	Bone
5	adenocarcinoma	IIA	2	1	Lymph node
6	adenocarcinoma	IIIB	4	2	Lymph node
7	mucinous adenocarcinoma	IIIB	2	2	Lymph node
8	adenosquamous cell carcinoma	IIIA	2	2	Bone
9	mucoepidermoid	IIA	2	0	Soft tissue

T, tumor; N, lymph nodes.

## Data Availability

Data will be made available on request to the corresponding author.

## Supplementary Materials

The following supporting information can be downloaded at: https://www.mdpi.com/article/10.3390/biom15010036/s1, Figure S1: C/EBPβ expression in primary and metastatic lung cancer tissues; Figure S2: C/EBPβ knockdown inhibited lung cancer metastasis; Figure S3: The expression of C/EBPβ varies in NSCLC cells lines; Figure S4: Confirmation of the efficiency of C/EBPβ expression changes and their correlation with cell apoptosis; Figure S5: A549 cells exhibited morphological changes in response to C/EBPβ expression under hypoxic conditions; Figure S6: The changes in migration and invasion induced by C/EBPβ expression are independent of cell proliferation; Figure S7: The expression of *HIF1A* and *SLC2A1* was reduced by C/EBPβ depletion under hypoxic conditions in the microarray data; Figure S8: Changes in the mRNA levels of *CEBPB*, *SLC2A1*, and *HIF1A* were confirmed after treatment with siNC, siHIF-1α, or siGLUT1 in NSCLC cells; Figure S9: Under normoxic conditions, HIF-1α does not affect the proliferation, migration, or invasion of NSCLC cells; Figure S10: The expression of *HIF1A* is correlated with *CEBPB* and *SLC2A1* in lung adenocarcinoma; Original images of Figure 2B,D,E can be found in Supplementary Materials.
