# Supplementary material for: C/EBPβ Regulates HIF-1α-Driven Invasion of Non-Small-Cell Lung Cancer Cells
Source: Biomolecules. 2024 Dec 30;15(1):36. doi: 10.3390/biom15010036 (PMC11764306; doi:10.3390/biom15010036)

Supplementary File

C/EBPβ regulates HIF-1α-driven invasion of Non-Small-Cell Lung Cancer Cells

Seung Hee Seo[1†], Ji Hae Lee[1†], Eun Kyung Choi[1], Seung Bae Rho[1] and Kyungsil Yoon[1*]

[1] Cancer Metastasis Branch, Research Institute, National Cancer Center, Goyang 10408, South Korea

[†] These authors contributed equally to this work

*** Correspondence: kyoon@ncc.re.kr**

Supplementary Figure S1.

C/EBPβ expression in primary and metastatic lung cancer tissues.

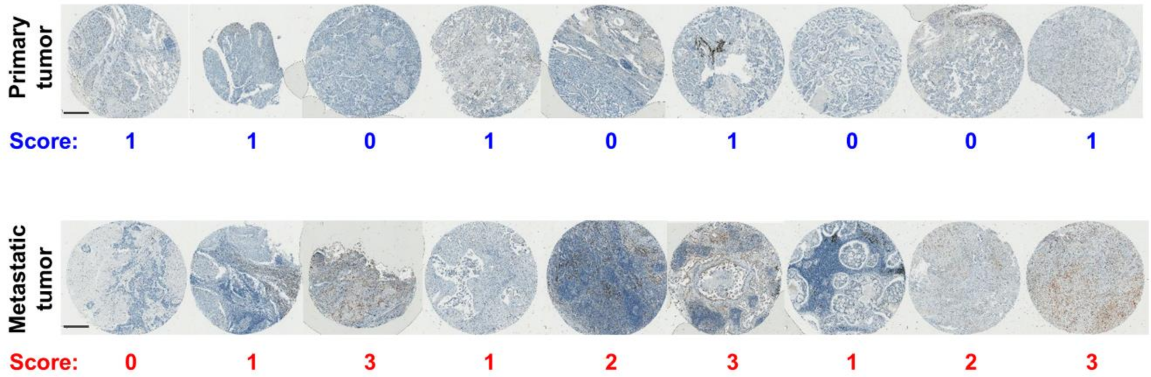

Images of metastatic tumors matched with primary tumors were captured using the Aperio ImageScope software. Tissue scores for each sample are shown below the images. Scale bars, 500 μm.

Supplementary Figure S2. C/EBPβ knockdown inhibited lung cancer metastasis.

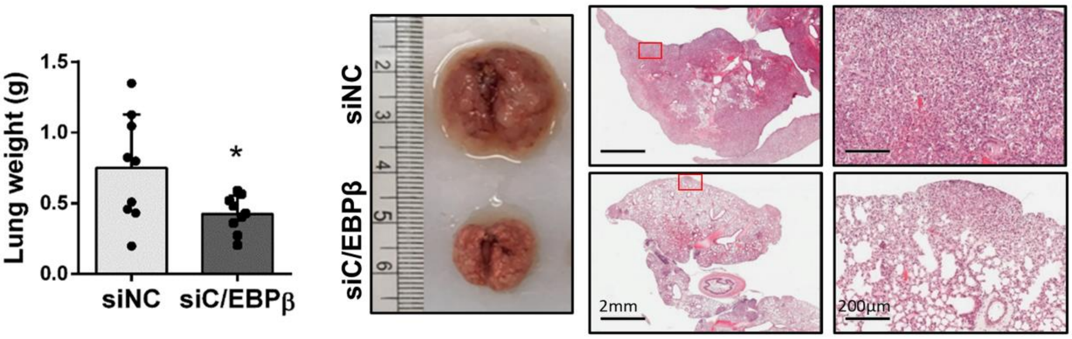

A549 cells (5 × 10^6) were intravenously injected into the tail vein of BALB/c nude mice. After nine weeks, the mice were anesthetized, and tumor-bearing lung weights were measured. H&E staining revealed that C/EBPβ knockdown significantly reduced metastatic tumor formation in the lungs.

Supplementary Figure S3. The expression of C/EBPβ varies in NSCLC cells lines.

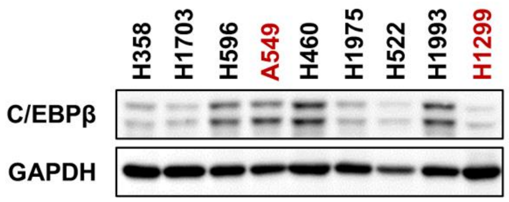

C/EBPβ protein levels in various NSCLC cell lines were determined by western blotting.

Supplementary Figure S4. Confirmation of the efficiency of C/EBPβ expression changes and their correlation with cell apoptosis.

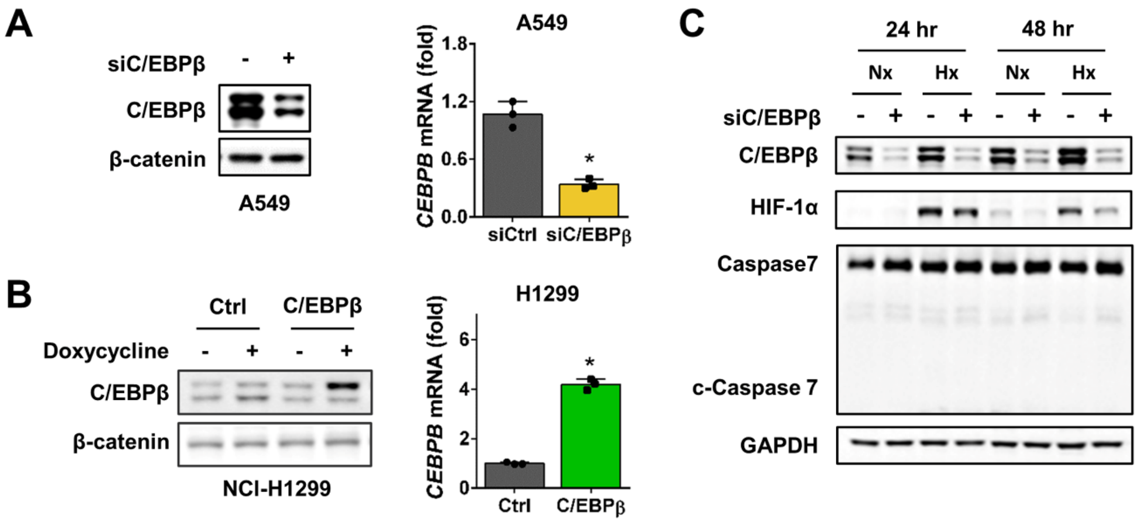

(A) A549 cells were transfected with 20 nM of siNC or siCEBPβ and protein and mRNA levels were confirmed by western blotting (left) and RT-PCR (right). (B) NCI-H1299-Tet-C/EBPβ or control cells were treated with 100 ng/ml doxycycline for 48 h and protein and mRNA levels were assessed by RT-qPCR and western blotting. (C) C/EBPβ-silenced A549 cells were exposed to hypoxia for 24 and 48 h under the same conditions used for the cell proliferation assay. Caspase 7, an apoptosis marker, was analyzed in relation to C/EBPβ reduction by Western blotting.

Supplementary Figure S5. A549 cells exhibited morphological changes in response to C/EBPβ expression under hypoxic conditions.

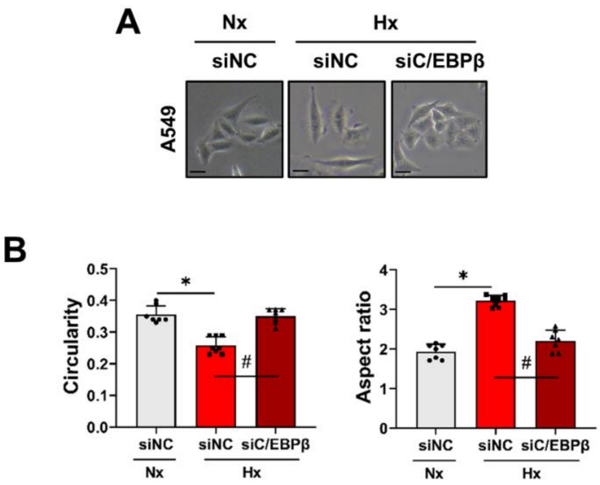

(A) A549 was transiently transfected with either siNC or siC/EBPβ. Changes in cell morphology were observed using bright-field optical microscopy at 8 h after exposure to 1% oxygen. Scale bar, 20 μm. (B) Images from each group were quantified using the cell descriptor plugin in ImageJ. Circularity and aspect ratio were used as quantitative parameters: Circularity = $(4\pi \times \text{Area}) / \text{Perimeter}^2$, AR = (Major axis length) / (Minor axis length)

Supplementary Figure S6. The changes in migration and invasion induced by C/EBPβ expression are independent of cell proliferation.

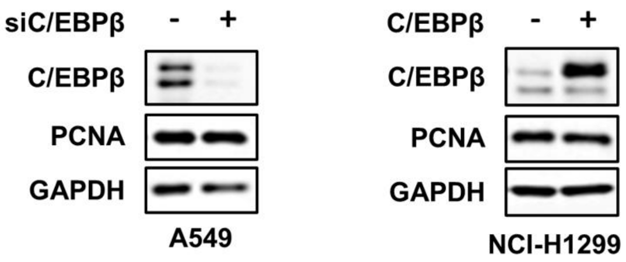

Western blot assays were conducted under the same conditions as those used for the trans-well assay in A549 and NCI-H1299. Proliferating cell nuclear antigen (PCNA), a proliferation marker, was analyzed in relation to changes in C/EBPβ levels.

Supplementary Figure S7. The expression of *HIF1A* and *SLC2A1* was reduced by C/EBPβ depletion under hypoxic conditions in the microarray data.

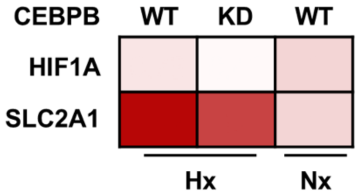

The microarray results were converted to fold change values relative to the normoxic condition in WT. The heatmap showed the expression changes of *HIF1A* and *SLC2A1* in C/EBPβ depleted A549 cells exposed to hypoxic conditions for 4 h.

Supplementary Figure S8. Changes in the mRNA levels of *CEBPB*, *SLC2A1*, and *HIF1A* were confirmed after treatment with siNC, siHIF-1α, or siGLUT1 in NSCLC cells.

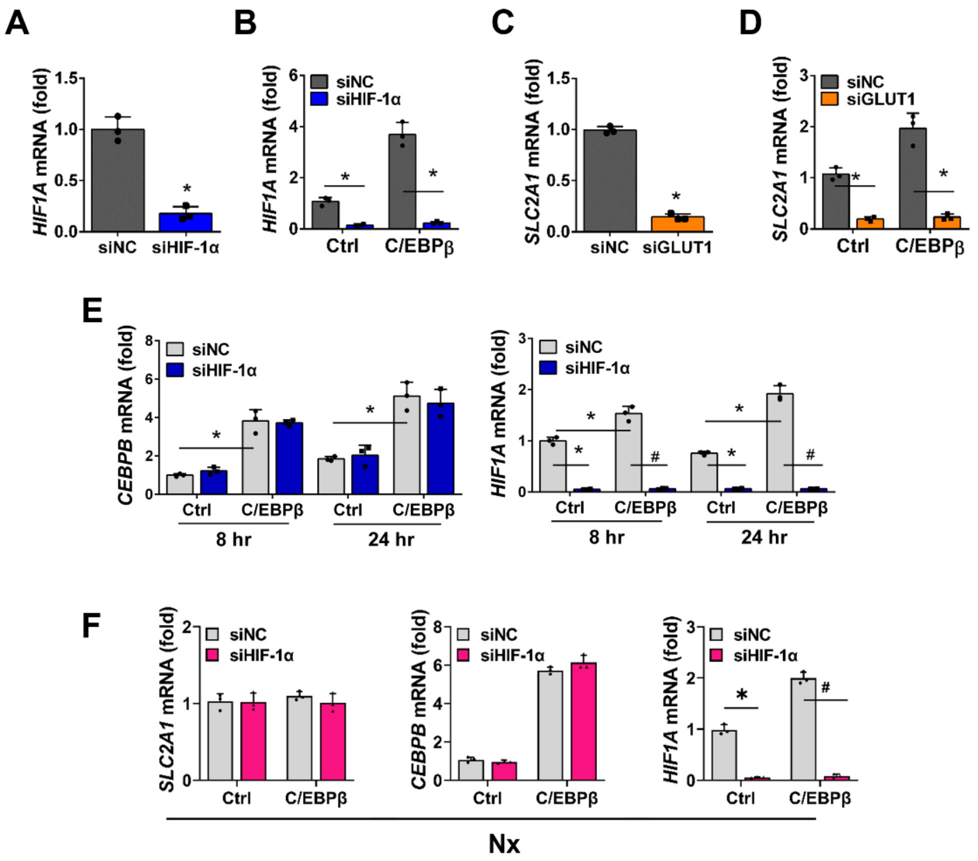

(A and C) A549 cells were transfected with siNC, siHIF-1α or siGLUT1 and knockdown efficiency was assessed by RT-qPCR. (B, D, and E) NCI-H1299-Tet-C/EBPβ cells were transfected with siNC, siHIF-1α, or siGLUT1 under hypoxic conditions and knockdown efficiency was assessed by RT-qPCR. (F) The mRNA levels of *SLC2A1*, *CEBPB*, and *HIF1A* were measured by RT-qPCR after transfection with siNC or siHIF-1α in NCI-H1299-Tet-C/EBPβ cells under normoxic conditions for 24 h. *$p < 0.05$, #$p < 0.05$

Supplementary Figure S9. Under normoxic conditions, HIF-1α does not affect the proliferation, migration, or invasion of NSCLC cells.

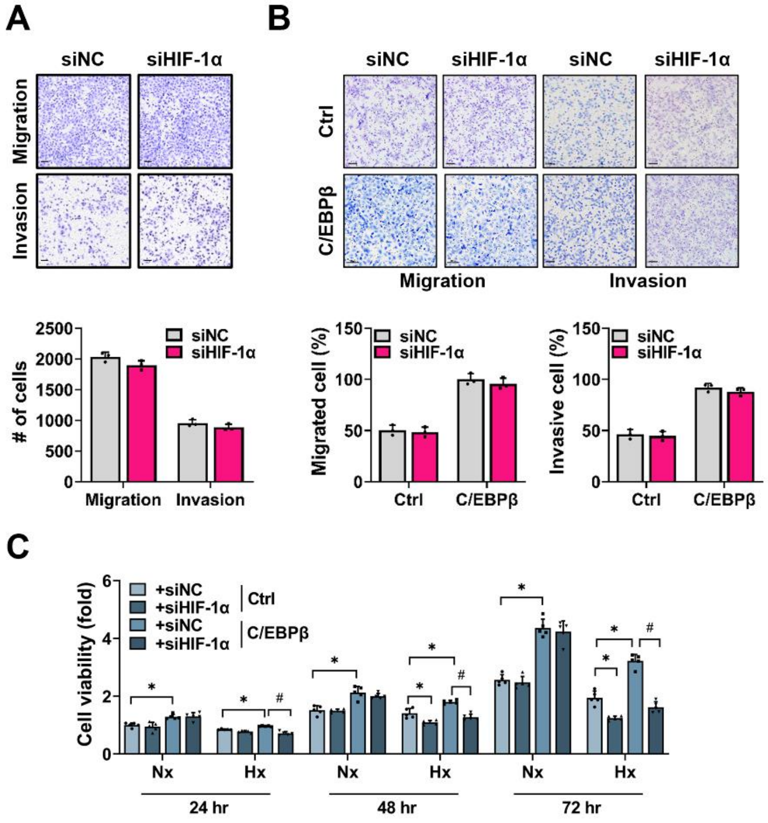

(A and B) Effects of HIF-1α knockdown on migration and invasion were evaluated in A549, NCI-H1299-Tet-control and NCI-H1299-Tet-C/EBPβ cells using a trans-well assay. Cells were seeded in trans-well inserts at 100% confluence in serum-free medium to avoid the influence of cell proliferation on the migration and invasion results. Scale bar, 20 μm. (A) A549 cells and (B) NCI-H1299 cells were maintained under normoxic conditions for 16 h for migration and invasion assay. (C) Cell proliferation was evaluated at 24-h intervals under normoxic and hypoxic conditions. *$p < 0.05$, #$p < 0.05$.

Supplementary Figure S10. The expression of *HIF1A* is correlated with *CEBPB* and *SLC2A1* in lung adenocarcinoma.

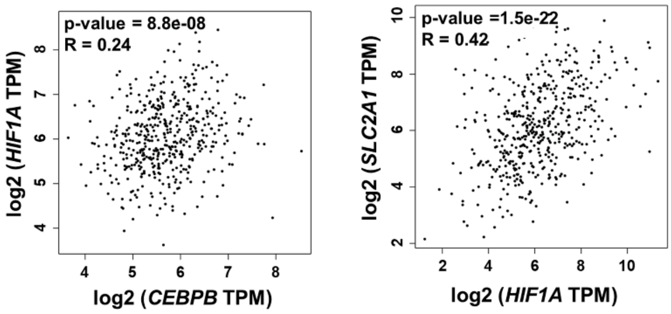

The *CEBPB-HIF1A* and *HIF1A-SLC2A1* correlation were analyzed in lung adenocarcinoma using TCGA database via GEPIA platform. Spearman correlation coefficients and p-values are displayed on each plot.

Original Western blot images - Figure 2B

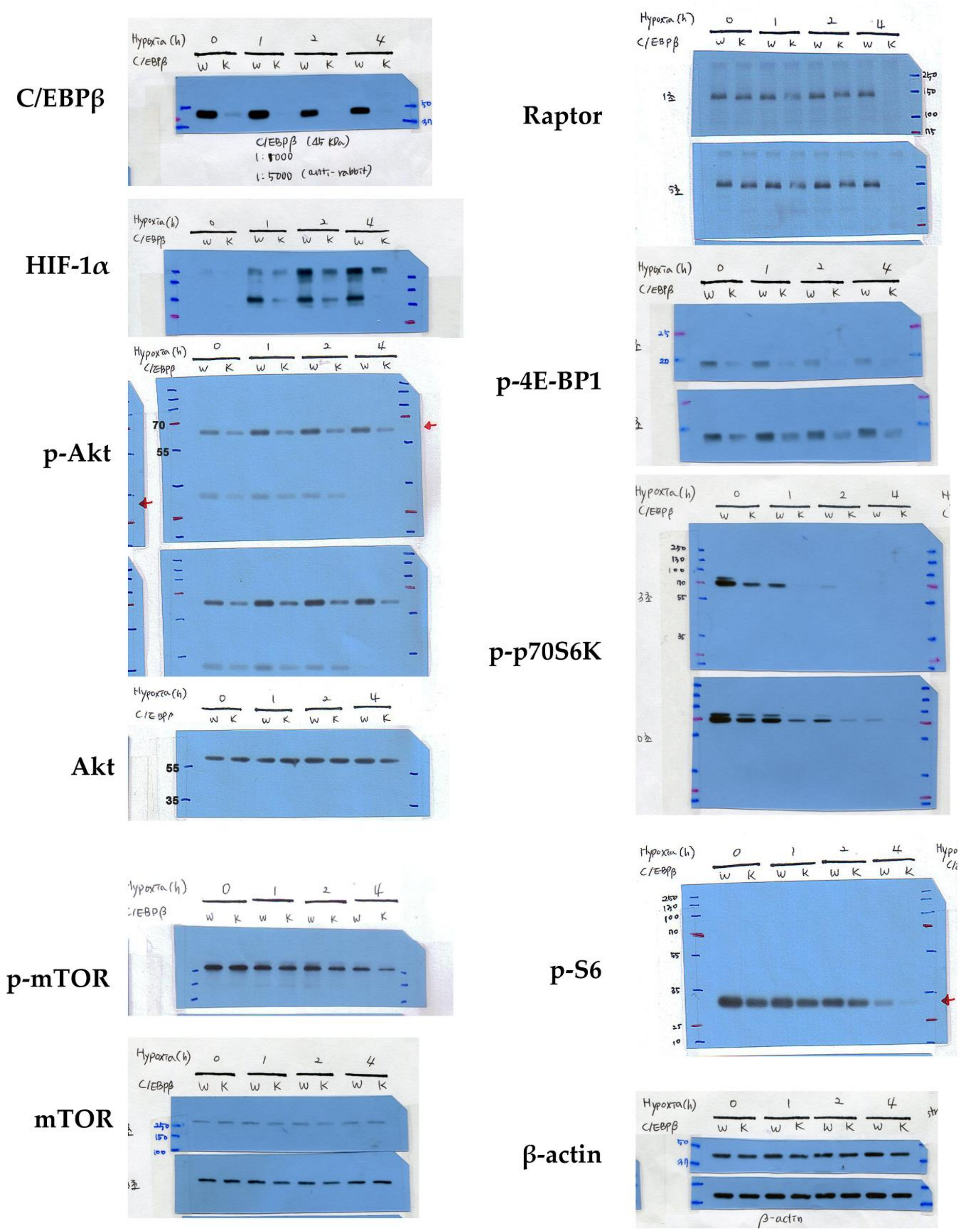

Original Western blot images - Figure 2D

A549

C/EBPβ

HIF-1α

GAPDH

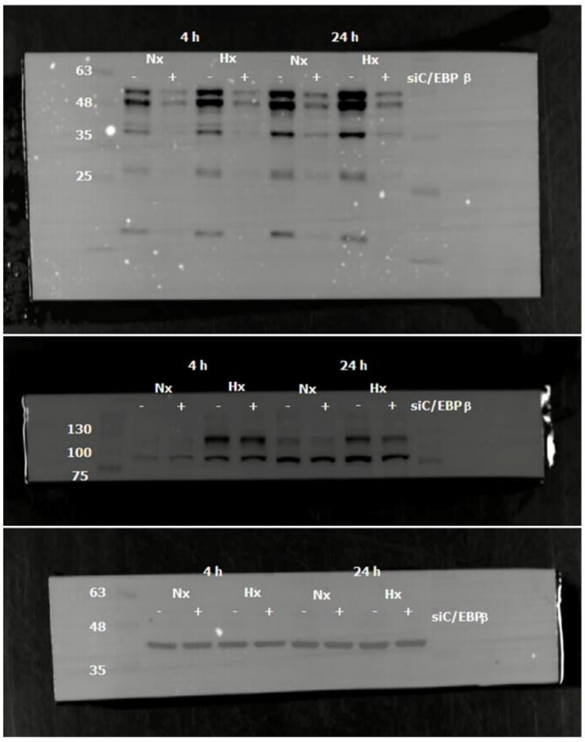

NCI-H1299

C/EBPβ

HIF-1α

GAPDH

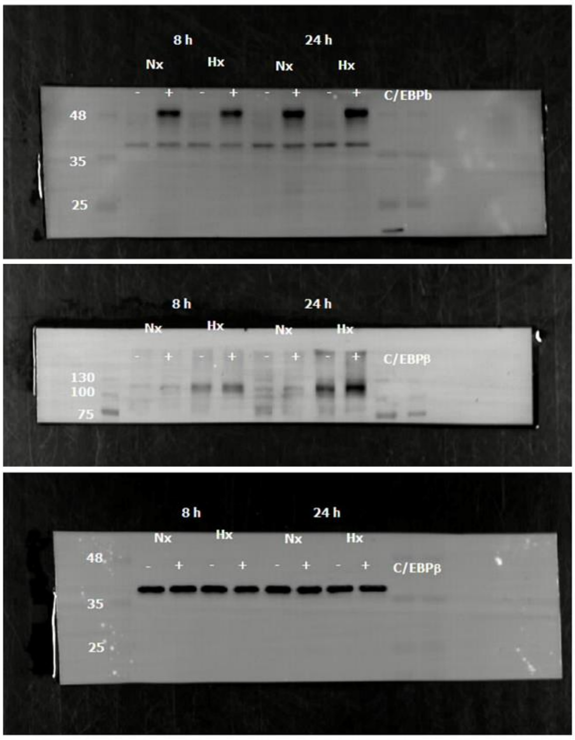

Original Western blot images - Figure 2E

HIF-1α

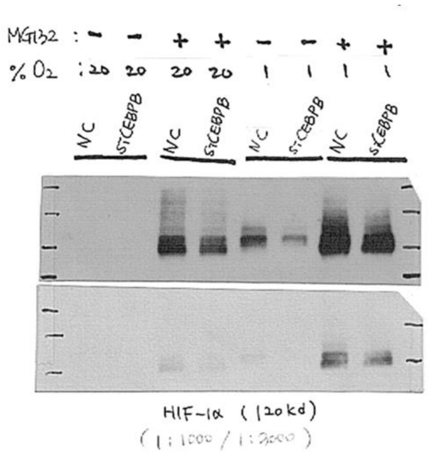

C/EBPβ

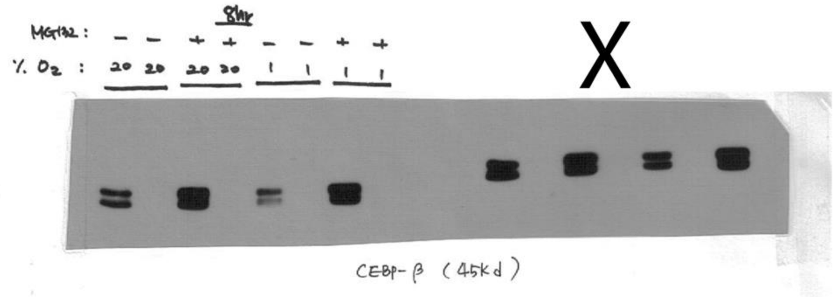

β-actin

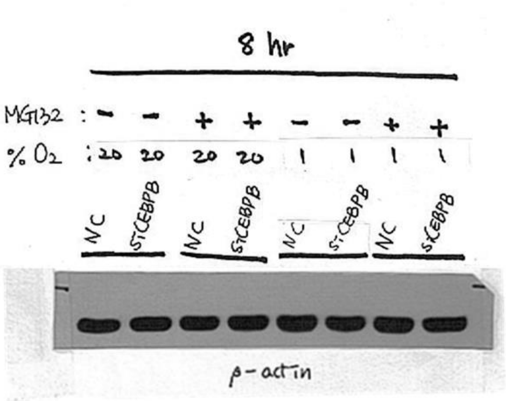

Supplement: Supplementary file 1 [file biomolecules-15-00036-s001.zip › biomolecules-3357945-supplementary.pdf]
